# Influence of helical pitch and gantry rotation time on image quality and file size in ultrahigh-resolution photon-counting detector CT

**DOI:** 10.1038/s41598-024-59729-6

**Published:** 2024-04-23

**Authors:** Philipp Feldle, Jan-Peter Grunz, Henner Huflage, Andreas Steven Kunz, Süleyman Ergün, Saif Afat, Philipp Gruschwitz, Lukas Görtz, Lenhard Pennig, Thorsten Alexander Bley, Nora Conrads

**Affiliations:** 1 Department of Diagnostic and Interventional Radiology, University Hospital Wuerzburg, Oberduerrbacher Straße 6, 97080 Wuerzburg, Germany; 2 Institute of Anatomy and Cell Biology, University of Wuerzburg, Koellikerstraße 6, 97070 Wuerzburg, Germany; 3 Department of Diagnostic and Interventional Radiology, University Hospital Tuebingen, Hoppe-Seyler-Str 3, 72076 Tuebingen, Germany; 4 Institute for Diagnostic and Interventional Radiology, Faculty of Medicine and University Hospital Cologne, University of Cologne, Kerpener Straße 62, 50937 Cologne, Germany

**Keywords:** Photon-counting, Tomography, x-ray computed, Helical pitch factor, Gantry rotation time, Raw data, Bone imaging, Radiography

## Abstract

The goal of this experimental study was to quantify the influence of helical pitch and gantry rotation time on image quality and file size in ultrahigh-resolution photon-counting CT (UHR-PCCT). Cervical and lumbar spine, pelvis, and upper legs of two fresh-frozen cadaveric specimens were subjected to nine dose-matched UHR-PCCT scan protocols employing a collimation of 120 × 0.2 mm with varying pitch (0.3/1.0/1.2) and rotation time (0.25/0.5/1.0 s). Image quality was analyzed independently by five radiologists and further substantiated by placing normed regions of interest to record mean signal attenuation and noise. Effective mAs, CT dose index (CTDI_vol_), size-specific dose estimate (SSDE), scan duration, and raw data file size were compared. Regardless of anatomical region, no significant difference was ascertained for CTDI_vol_ (p ≥ 0.204) and SSDE (p ≥ 0.240) among protocols. While exam duration differed substantially (all p ≤ 0.016), the lowest scan time was recorded for high-pitch protocols (4.3 ± 1.0 s) and the highest for low-pitch protocols (43.6 ± 15.4 s). The combination of high helical pitch and short gantry rotation times produced the lowest perceived image quality (intraclass correlation coefficient 0.866; 95% confidence interval 0.807–0.910; p < 0.001) and highest noise. Raw data size increased with acquisition time (15.4 ± 5.0 to 235.0 ± 83.5 GByte; p ≤ 0.013). Rotation time and pitch factor have considerable influence on image quality in UHR-PCCT and must therefore be chosen deliberately for different musculoskeletal imaging tasks. In examinations with long acquisition times, raw data size increases considerably, consequently limiting clinical applicability for larger scan volumes.

## Introduction

Computed tomography scans of the spine, pelvis, and upper thighs bear considerably greater image information compared to conventional radiography, which is especially relevant in cases of pathologically altered bone matrix and consecutive osteoporotic fractures commonly seen in elderly patients^1^. Due to a steady increase in the population’s age, CT imaging oftentimes represents the imaging modality of choice in suchlike scenarios^2,3^. With the increasing availability of photon-counting CT (PCCT) and its inherent potential for ultrahigh-resolution (UHR) imaging without additional dose penalty, this type of examination is likely to become even more prevalent. Recent studies have shown that improved image quality and reduced radiation exposure can be achieved by using UHR mode with PCCT systems^4–7^. While dose modulation based on topograms has become a standard in modern CT scanners, certain adjustments such as gantry rotation time, helical pitch factor, and tube voltage level must still be specifically selected. Contrary to conventional energy-integrating detector CT systems, which typically operate at lower kV values to optimize image contrast, modern PCCT scanners benefit from a higher kV value for optimal spectral separation.

In UHR acquisition mode, the only commercially available PCCT system at present reduces collimation from 144 × 0.4 mm in standard mode to 120 × 0.2 mm^8^. While musculoskeletal imaging in particular benefits from superior spatial resolution, raw data of UHR-PCCT are substantially larger than comparable PCCT files acquired in standard mode or on prior scanner generations with energy-integrating detector technique. This often results in delayed image reconstruction and fully engaged data storage, which limits patient throughput in clinical routine. Since the helical pitch factor is dependent on the detector collimation, the table feed is reduced with thinner collimation, which in turn leads to increased examination times if all other parameters remain constant^9,10^. Notably, the vendor-specific “effective tube current” is also affected by collimation, pitch factor and rotation time, consecutively influencing image quality^9–12^.

With multiple interdependencies between scan parameters, precise knowledge of the individual effects is key to establish optimized examination protocols in UHR-PCCT. However, as of this writing, PCCT literature is primarily focused on comparisons to energy-integrating detector CT, whereas aspects like helical pitch and gantry rotation time have not been thoroughly assessed thus far. In order to address this knowledge gap, the aim of this experimental study was to quantify the influence of both factors on image quality and raw data file size in musculoskeletal UHR-PCCT.

## Material and methods

### Study sample

Permission for this experimental single-center study was granted by the ethics committee of the University of Würzburg (Germany). Two fresh-frozen cadaveric specimens with a body mass index of 27 and 29 kg/m^2^, respectively, were obtained from our university’s anatomical institute. As both body donors had agreed to posthumous scientific research during their respective lifetimes, no further written informed consent was required. The study was planned and executed in accordance with institutional regulations, as well as state and federal laws.

### Imaging

Refraining from repositioning in the meantime, the cervical and lumbar spine, as well as the pelvis and upper legs of specimens were subjected to a total of nine dose-matched scan protocols with varying acquisition parameters in June 2023. Scans were planned on the corresponding scout images, and it was ensured what identical scan lengths were used for all consecutive acquisitions. This approach ensured consistency throughout the study, maintaining uniformity in the scan regions throughout the acquisitions. All examinations were performed on a first-generation cadmium-telluride-based PCCT scanner (Naeotom Alpha, Siemens Healthcare). Employing a collimation of 120 × 0.2 mm in UHR mode and a tube potential of 140 kVp, automatic tube current modulation (CARE keV) was activated as per clinical routine for each anatomical region. The vendor-specific dimensionless image quality marker (formerly referred to as ‘reference mAs’) was set to 30. Scan volumes were adapted to the clinical standard for each region, and scan lengths were identical among the different protocols. Effective mAs, volume CT dose indices (CTDI_vol_), size-specific dose estimates (SSDE), scan durations, and respective raw data file sizes were recorded for each examination. Scan protocols were characterized by nine unique combinations of gantry rotation time (0.25, 0.5 or 1.0 s) and helical pitch factor (0.3, 1.0 or 1.2). Higher or lower pitch factors were not applicable due absolute tube current capacities. Protocol-specific scan parameters are presented in Table 1. For image reconstruction in UHR mode, the “fixed axial mode” was selected with slice thickness and increment both set to 0.6 mm. Although the maximum resolution of the investigated scanner is even higher than 0.6 mm, the so-called "small pixel effect" allows for considerable noise reduction advantages in the reconstruction of UHR data below the raw system MTF. Depending on the applied radiation dose, the use of thinner slices could lead to considerable disadvantages in terms of image noise^7,13^. Raw data was reconstructed using a dedicated ultrasharp bone kernel (Br80; ρ_50_ = 19.31 line pairs/cm; ρ_10_ = 24.88 lp/cm) with iterative reconstruction (Quantum Iterative Reconstruction [QIR], Siemens Healthcare) set to strength level 2^14^. Reconstruction matrix was automatically adjusted by the scanner for optimal image quality in each anatomical region, resulting in image matrices of 768 to 1024 pixels.

**Table 1:** Scan parameters.

Parameter	P1	P2	P3	P4	P5	P6	P7	P8	P9
Tube voltage [kVp]	140	140	140	140	140	140	140	140	140
Mean eff. tube current [mAs]	52.9 ± 12.7	52.5 ± 12.3	52.0 ± 11.4	52.6 ± 12.3	52.5 ± 12.3	52.6 ± 12.3	53.4 ± 11.5	12.5 ± 12.3	52.8 ± 12.2
Collimation [mm]	120 × 0.2	120 × 0.2	120 × 0.2	120 × 0.2	120 × 0.2	120 × 0.2	120 × 0.2	120 × 0.2	120 × 0.2
Pitch factor	0.3	1.0	1.2	0.3	1.0	1.2	0.3	1.0	1.2
Gantry rotation time [sec]	0.25	0.25	0.25	0.5	0.5	0.5	1.0	1.0	1.0
Mean CTDI_vol_ [mGy]	6.4 ± 1.5	6.3 ± 1.3	6.2 ± 1.2	6.2 ± 1.4	6.2 ± 1.3	6.2 ± 1.3	6.3 ± 1.3	6.2 ± 1.3	6.2 ± 1.3
Mean SSDE [mGy]	9.2 ± 1.6	9.0 ± 1.4	9.0 ± 1.3	9.0 ± 1.4	9.0 ± 1.5	9.0 ± 1.4	9.1 ± 1.4	9.0 ± 1.5	9.0 ± 1.4

*CTDI*_*vol*_ volume CT dose index, *P1–9* scan protocol 1–9, *SSDE* size-specific dose estimate.

### Image quality analysis

For subjective image quality assessment, five radiologists with 4 to 14 years of experience in clinical CT imaging evaluated all datasets independently and blinded to any scan protocol-related information in July 2023. Image quality was analyzed individually based on a seven-point rating scale (1: insufficient, 2: poor, 3: fair, 4: moderate, 5: good, 6: very good, 7: excellent). To provide an additional objective criterion of image quality, a radiologist with 8 years of CT imaging experience placed normed regions of interest (ROI) in subcutaneous fat within each anatomical region. Carefully avoiding partial volume effects and artifacts by osteosynthesis material, ROIs were manually positioned in datasets generated with protocol P1 and then copied to identical locations in scan protocols P2–P9. Of note, no helical artifacts were encountered, especially not in the subcutaneous fat tissue where ROIs were positioned. For each ROI, mean signal attenuation and its standard deviation were recorded in Hounsfield units (HU). The signal standard deviation in lipid tissue was considered representative of image noise due to fat possessing a more homogenous texture than muscle or bone.

### Statistics

Statistical analyses were conducted using SPSS Statistics 28 software (IBM, Armonk, USA). For parametric variables, data analysis involved assessing normal distribution using the Shapiro–Wilk test and conducting repeated measures ANOVA for normally distributed parametric variables. To examine differences in subjective image quality, Friedman's rank-based analysis of variance was used for rating comparisons among the various scan protocols. Bonferroni-corrected pairwise post-hoc tests were performed to account for multiple comparisons. To determine the level of interrater reliability, the intraclass correlation coefficient was calculated using a two-way random effects model for absolute agreement. Statistical significance was set at p ≤ 0.05.

## Results

### Examination characteristics

For cervical spine scans, CTDI_vol_ and SSDE ranged between 5.5–5.8 mGy and 8.7–9.2 mGy, respectively. CTDI_vol_ (7.8–8.2 mGy) and SSDE (9.9–10.3 mGy) ranges for lumbar spine examinations were slightly higher. For pelvis and upper leg studies, CTDI_vol_ was measured between 6.4–6.6 mGy and 5.0–5.2 mGy, while corresponding SSDE ranges were established between 8.7–9.0 mGy and 8.3–8.5 mGy. Regardless of anatomical region, no significant difference was ascertained for CTDI_vol_ (p ≥ 0.204) or SSDE (p ≥ 0.240) among any pair of scan protocols. Indicators of dose burden associated with each setting are included in Table 1. Despite comparable radiation exposure, the examination time associated with each protocol differed substantially (all p ≤ 0.016). The lowest average scan time was recorded for the high-pitch protocol P3 (4.3 ± 1.0 s), whereas the highest scan duration was determined for the low-pitch protocol with the slowest gantry rotation time (P7; 43.6 ± 15.4 s). Notably, raw data file size increased with overall acquisition time, resulting in P7 incurring the largest (235.0 ± 83.5 GByte) and P3 incurring the smallest raw datasets (15.4 ± 5.0 GByte). Overall raw data file size differed considerably among protocols (p ≤ 0.013; Table 2).

**Table 2:** Scan duration and raw data file size.

Parameter	P1	P2	P3	P4	P5	P6	P7	P8	P9
Scan duration [sec]	11.3 ± 3.9	4.7 ± 1.2	4.3 ± 1.0	22.0 ± 7.7	7.7 ± 2.3	6.7 ± 1.9	43.6 ± 15.4	14.5 ± 4.6	12.3 ± 3.9
Raw data file size [GByte]	56.0 ± 19.9	18.2 ± 6.0	15.4 ± 5.0	117.5 ± 41.8	38.1 ± 12.5	32.2 ± 10.4	235.0 ± 83.5	76.2 ± 25.1	64.3 ± 20.9

*P1–9* scan protocol 1–9.

### Subjective image quality

**Figure 1:**
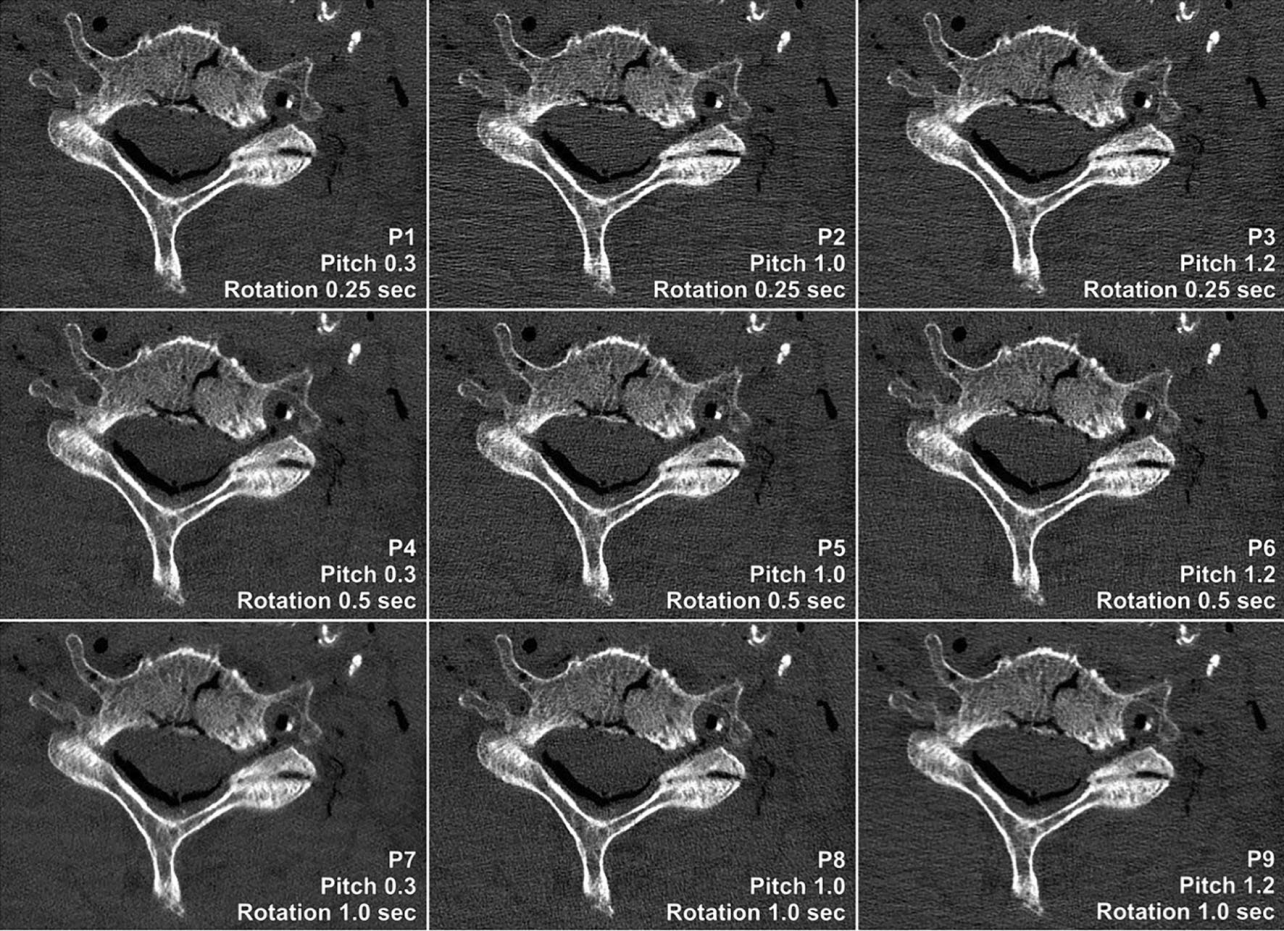
Ultrahigh-resolution photon-counting CT of the cervical spine with different combinations of helical pitch factor and gantry rotation time. Note the pronounced differences in image noise and sharpness among the nine scan protocols despite comparable radiation doses (5.71–5.94 mGy).

**Figure 2:**
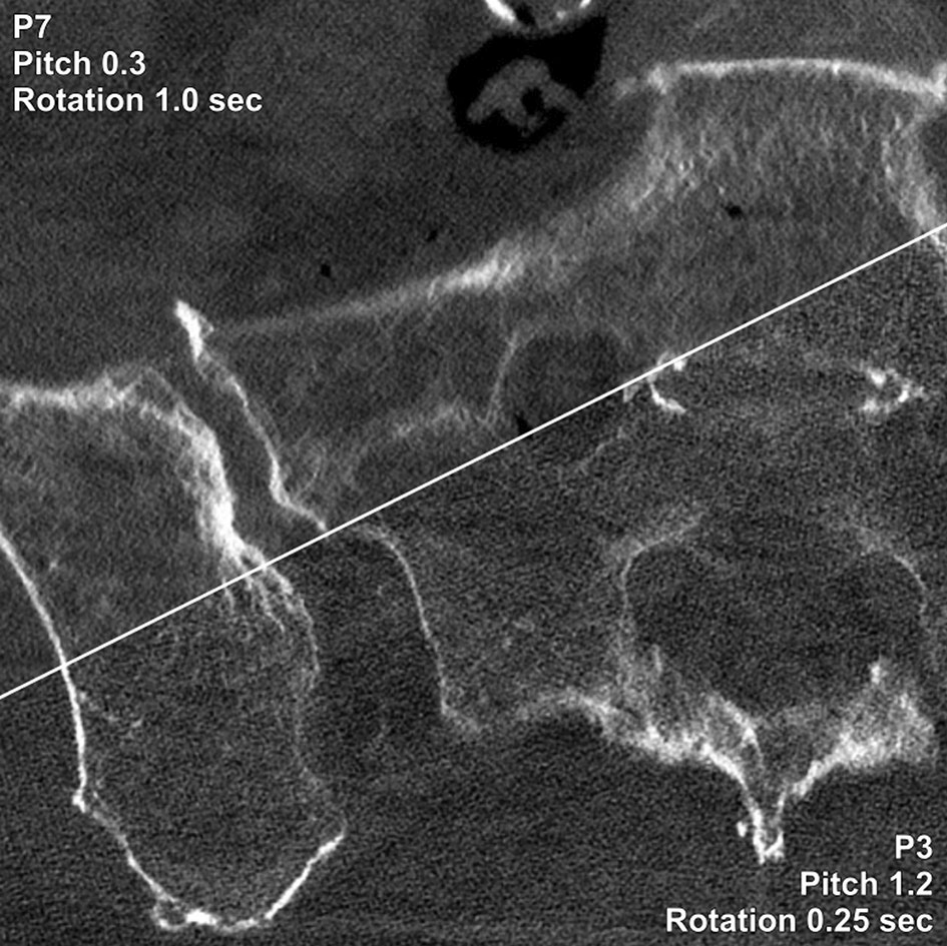
Image quality comparison between a low pitch/slow gantry rotation (P7) and high pitch/fast gantry rotation (P3) protocol in a pelvic photon-counting CT scan. Scan duration (P7: 35.6 vs. P3: 3.8 s) and raw data file size (191.4 vs. 12.8 GByte) differed substantially, whereas CTDI_vol_ was identical (5.83 vs. 5.83 mGy).

Figure 1 comprises a compilation of cervical spine studies acquired with all nine scan protocols in UHR mode. The combination of a high helical pitch factor and a short gantry rotation time (P3) resulted in the lowest perceived image quality among scan protocols (median score 3, interquartile range 3–4). While no significant differences were established between protocols P1, P2, and P4–P9 (p ≥ 0.759), each individual setting received superior image quality ratings compared to P3 (all p < 0.001). A side-by-side comparison between high-pitch/fast gantry rotation (P3) and low-pitch/slow gantry rotation (P7) is provided in Fig. 2. Interrater agreement regarding image quality was good, as indicated by an intraclass correlation coefficient of 0.866 (95% confidence interval 0.807–0.910; p < 0.001).

### Objective image quality

**Figure 3:**
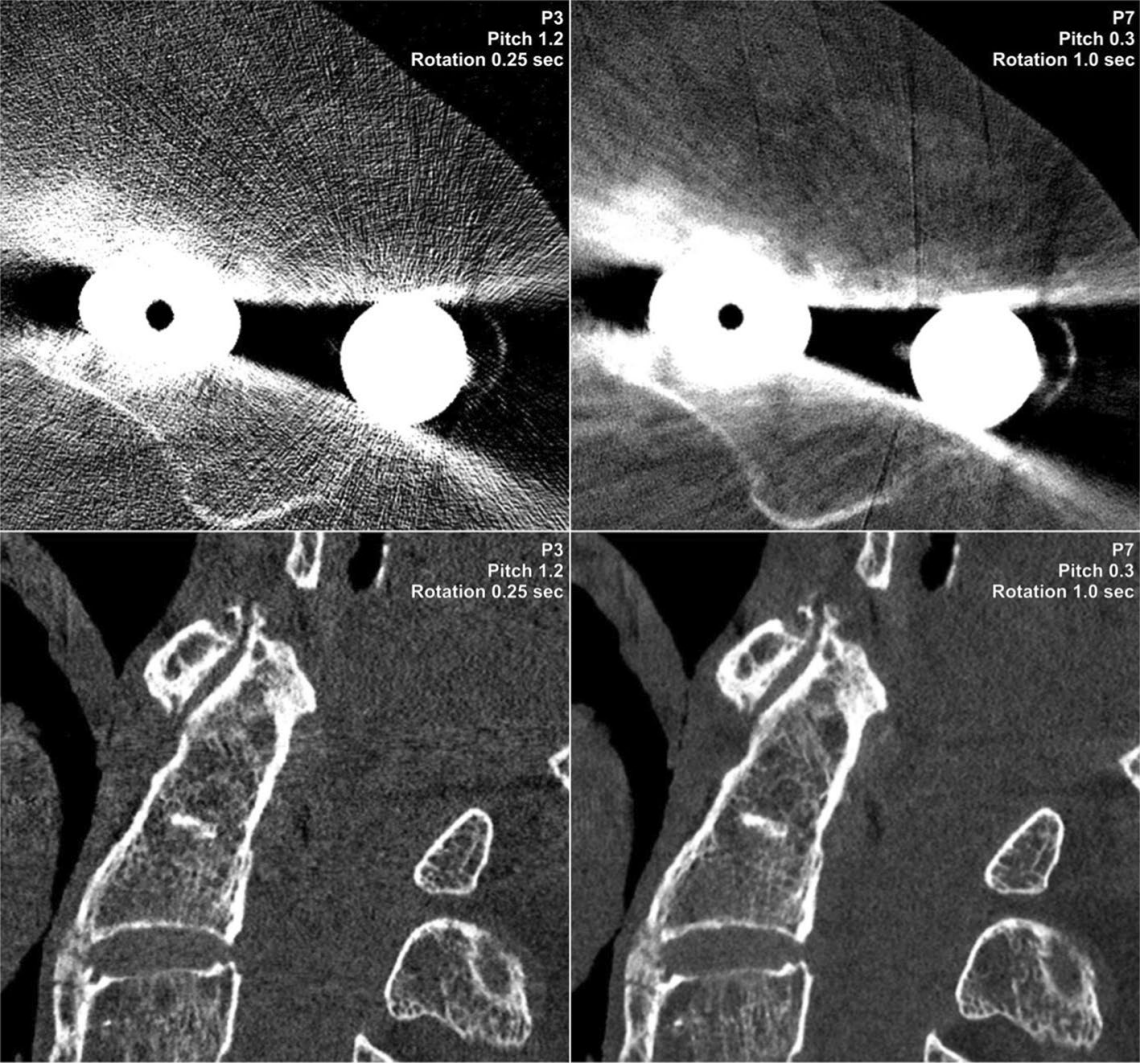
Image noise increases considerably when combining a high pitch factor with fast gantry rotation time (P3). This effect becomes even more apparent in the presence of metal artifacts. In contrast, employing a low pitch factor with slow gantry rotation time (P7) partially offset the noise increase in a cadaveric specimen with upper leg osteosynthesis (*upper row*), while basically removing the streak artifacts in a body donor with dental implants (*lower row*).

To account for differences in signal characteristics, image noise in subcutaneous lipid tissue was compared among scan protocols. The highest noise level was recorded for the high-pitch/fast rotation protocol P3 (159.1 ± 16.6 HU), while the noise magnitude was lowest in the low-pitch/slow rotation protocol P7 (85.1 ± 10.4 HU). The image noise ascertained in P3 was considerably higher than in all three scan protocols with a gantry rotation time of 1 s (P7–9; p ≤ 0.017). Apart from P4 (104.1 ± 16.5 HU; p = 0.120), the image noise measured in P7 was substantially lower than in all other scan protocols (p ≤ 0.027), despite comparable radiation dose. Noise measurements and subjective image quality ratings are summarized in Table 3. Figure 3 illustrates a comparison between the noise level of high pitch/fast rotation (P3) vs. low pitch/slow rotation protocols (P7) in the presence of different metal implants.

**Table 3:** Image quality analysis.

Parameter	P1	P2	P3	P4	P5	P6	P7	P8	P9
Median subjective image quality	5 (4–5)	5 (4–6)	3 (3–4)	5 (4–6)	5 (4–6)	5 (4–6)	5 (4–6)	5 (4–6)	5 (4–6)
Mean image noise [HU]	119.8 ± 13.1	145.9 ± 22.4	159.1 ± 16.6	104.1 ± 16.5	134.2 ± 19.3	154.4 ± 22.4	85.1 ± 10.4	120.0 ± 18.5	128.2 ± 17.4

*P1–9* scan protocol 1–9.

## Discussion

This experimental study analyzed the influence of helical pitch and gantry rotation time on image quality and raw data file size in unenhanced musculoskeletal examinations with a first-generation photon-counting detector CT system in ultrahigh-resolution mode. The lowest average scan time was recorded for the high-pitch protocol with the shortest gantry rotation time and the highest scan duration was determined for the low-pitch protocol with the slowest gantry rotation time. However, regardless of the examined anatomical region, radiation dose exposure was comparable among all nine scan protocols. While the shortest scan time resulted in the smallest raw data file size, the respective combination of high helical pitch factor (1.2) and a short gantry rotation time (0.25 s) was also associated with the lowest perceived image quality and highest measured noise. As a major finding, no significant image quality differences were established between the other eight combinations of helical pitch and gantry rotation speed, suggesting potential for considerable scan time saving and data file size reduction.

Whereas UHR scan mode by definition offers a significantly higher spatial resolution with improved image quality over standard mode, the associated potential for radiation dose reduction is a novel feature of PCCT systems, which do not require dose-increasing comb or grid filters to modify the detector aperture^15,16^. However, since detector collimation is related to the helical pitch factor, UHR imaging incurs increased scan times compared with standard mode (120 × 0.2 mm vs. 144 × 0.4 mm) due to reduced table feed at identical pitch settings^9,10,15^. Consistent with current literature on energy-integrating CT and PCCT systems, our results indicate that selection of high pitch and short rotation time indeed reduces overall scan duration, albeit at the cost of impaired image quality^17^. Simultaneously, as a result of the interdependent relationship between effective mAs, tube current, rotation time and pitch $(Effective\, mAs=\frac{\mathrm{mA }\times \mathrm{ rotation\, time}}{{\text{pitch}}})$, merely altering the latter two will not affect the CTDI_vol_ or SSDE, as the effective mAs remains unchanged^18^. Specifically, with a small pitch and a high rotation time, the tube current can be kept relatively low, which prolongs the scanning process, but burden on the anode is reduced as there is no need to operate at its maximum settings^7^. This modification of settings is advantageous when higher doses are required, e.g., to realize higher image quality in adipose patients.

The presented results indicate, in concordance with prior studies on energy-integrating-CT, that a low pitch with constant rotation time or a low rotation time at constant pitch both minimize image noise, which is to be considered advantageous, e.g., with regards to artifact reduction^10,19^. However, due to the resulting extended scan time, adequate patient compliance is mandatory. Suchlike compliance would be particularly crucial for examinations with strong and poorly controllable motion sequences such as cardiac or, in certain cases, respiratory movement^20^. Of note, extended scan times are usually of questionable relevance in musculoskeletal imaging, where the reduction of metal artifacts is often more decisive, e.g., after implantation of pedicle screws or a total hip replacement. While not in the scope of the present study, recent PCCT publications have revealed that different metal artifact reduction algorithms and the use of virtual monoenergetic images bear the capability of effectively reducing metal artifacts^21–24^. Future studies are warranted to investigate whether additional modifications of helical pitch and gantry rotation speed can maximize this effect.

The combination of UHR scan mode, low pitch, and high gantry rotation time provides an increased number of projections and thus considerably larger raw data files for image reconstruction but at the same time reduces noise and consequently enhances image quality^9,17^. Partially offsetting the limitations associated with enormous file sizes, the z-axis collimation is generally reduced in UHR-PCCT^25^, which helps to concur with data transfer limits. On the other hand, Stein et al. made evident that new data transfer, storage, and processing solutions are needed to take full advantage of PCCT capabilities, such as improved spatial resolution and quantitative image post-processing capabilities even with a larger matrix^26^.

### Limitations

Some limitations of this experimental cadaveric study need to be mentioned: First, two specimens represent a small study population with correspondingly little variance in terms of body constitution, which may have impacted the study results. Second, due to the variable soft tissue content, standardized scan parameters along the entire spine were not applicable as image quality, e.g., of the cervicothoracic junction, would suffer massively compared to the upper cervical spine in the vicinity of the shoulder girdle^27^. Third, since metal implants occurred as incidental findings in the present study and were neither an inclusion nor exclusion criterion, only a descriptive statement regarding metal artifact severity is possible with the data collected. Of note, ultrasharp bone kernels for UHR-PCCT are currently not compatible with dedicated metal artifact reduction algorithms or virtual monoenergetic imaging^28^. In consequence, further studies are needed to evaluate the optimal scan parameters for metal artifact reduction with PCCT in UHR mode. Fourth, while noise texture plays an important role in image quality analysis, this study was primarily focused on assessing the relationship between raw data file size and helical pitch/gantry rotation time. With that being said, the evaluation of noise texture in future studies could be a valuable addition to provide a more holistic assessment of CT image quality. Fifth, the significant increase in file size seen in low-pitch protocols in combination with slow gantry rotation times must be addressed as a limitation with regards to routine clinical practice. Finally, only 140 kVp protocols were compared in the present study, since the broader spectrum (compared with e.g. 120 kVp) is considered superior for virtual monoenergetic reconstructions, which are commonly performed for clinical routine imaging^29,30^.

## Conclusion

Rotation time and pitch factor have a considerable influence on image quality in UHR-PCCT and must therefore be chosen deliberately for different musculoskeletal imaging tasks. In examinations with long acquisition times, raw data file size increases considerably, consequently limiting clinical applicability for larger scan volumes.

## Data Availability

The datasets generated and/or analyzed during this study are not publicly available as DICOM headers contain patient information. Data can be obtained on reasonable request from the corresponding author.

## Abbreviations

CTDI_vol_: volume computed tomography dose index
PCCT: Photon-counting computed tomography
ROI: region of interest
SSDE: size-specific dose estimate
UHR: ultrahigh-resolution
